# Comparative Pathogenicity and Transmissibility of the H7N9 Highly Pathogenic Avian Influenza Virus and the H7N9 Low Pathogenic Avian Influenza Virus in Chickens

**DOI:** 10.3390/v11111047

**Published:** 2019-11-10

**Authors:** Hao Yu, Kunpeng Zhang, Xumeng Ye, Wenqing Wang, Wenbo Wu, Xia Wang, Yun Guan, Zhuoliang He, Yong Wang, Peirong Jiao

**Affiliations:** 1 College of Veterinary Medicine, South China Agricultural University, 483 Wushan Road, Tianhe District, Guangzhou 510642, Chinawenbo0816@foxmail.com (W.W.); hezhuoliang@foxmail.com (Z.H.); 2 Department of Epidemiology, Public Health College, Harbin Medical University, Harbin 150081, China

**Keywords:** H7N9, avian influenza virus, pathogenicity, transmissibility, chickens

## Abstract

There were five outbreaks of H7N9 influenza virus in humans in China since it emerged in 2013, infecting >1000 people. The H7N9 low pathogenic influenza virus was inserted into four amino acids in the HA protein cleavage site to mutate into the H7N9 highly pathogenic virus. This emerging virus caused 15 outbreaks in chickens from the end of 2016 to date. Two H7N9 avian influenza virus (AIV) strains, A/chicken/Guangdong/A46/2013 (LPAIV) and A/chicken/Guangdong/Q29/2017 (HPAIV), were selected to compare the pathogenicity and transmissibility between H7N9 LPAIVs and HPAIVs in chickens. We inoculated 3- to 4-week-old specific-pathogen-free (SPF) chickens with 6 log_10_EID_50_/0.1 mL viruses via the ocular-nasal route and co-housed four chickens in each group. The inoculated chicken mortality rate in the A46 and Q29 groups was 1/5 and 5/5, respectively. Q29 virus replication was more efficient compared to the A46 virus in inoculated chickens. Infected chickens initiated viral shedding to naïve contact chickens through respiratory and digestive routes. Both viruses transmitted between chickens by naïve contact, but the Q29 virus had a higher pathogenicity in contact chickens than the A46 virus. Compared with early H7N9 LPAIVs, the pathogenicity and transmissibility of the emerging H7N9 HPAIV was stronger in chickens, indicating that H7N9 influenza virus may continue to threaten human and poultry health.

## 1. Introduction

Avian influenza is an infectious disease that is caused by avian influenza virus (AIV) and it infects poultry and various species of wild birds. AIV is a single-stranded negative-sense RNA virus belonging to the family *Orthomyxoviridae* and genus *Influenzavirus* A [1]. The virus contains eight gene segments encoding at least ten proteins, such as hemagglutinin (HA), neuraminidase (NA), polymerase acid (PA), polymerase basic 1 (PB1), PB2, nucleoprotein (NP), nonstructural 1 (NS1), NS2, matrix 1 (M1), and M2 [2]. To date, 16 HA and nine NA subtypes of AIVs have been detected in wild birds and poultry all over the world [1,3,4,5]. According to the pathogenicity of the virus in chickens, AIV is classified into the highly pathogenic avian influenza virus (HPAIV) and the low pathogenic avian influenza virus (LPAIV).

In February 2013, a new H7N9 influenza virus first infected a man in Shanghai, China and was identified as a low pathogenic influenza virus [6,7,8]. As of the November 2013, 142 human cases of H7N9 virus infection were reported to the WHO and 45 of these patients died [9]. The H7N9 low pathogenic influenza viruses were triple-reassortant viruses with six internal genes that originated from the local H9N2 AIVs, and the HA and NA genes originated from unknown H7 and N9 AIVs [10]. However, no clinical signs or deaths were seen in chickens infected with a new H7N9 AIV from birds which shared high homology across all eight gene segments with the human-origin viruses that killed humans, suggesting that the new H7N9 AIV may be the source of human infection [11,12]. Along with the virus spreading from the Yangtze River Delta region, the origin of the H7N9 influenza virus moved southward to the Pearl River Delta region of Guangdong Province in 2013, and two lineages have gradually formed: the Yangtze River Delta lineage and the Pearl River lineage [13]. Although the virus has spread from the two regions to other provinces, the Yangtze River Delta lineage is still the larger cluster [14].

From November 2013 to the end of 2016, H7N9 had over three waves in China. In June 2016, four amino acids from H7N9 LPAIVs were inserted into the HA protein cleavage site to mutate it into the H7N9 HPAIVs [15]. Since the end of 2016, 15 outbreaks of H7N9 avian influenza have occurred. The viruses caused 189,405 infected cases and 128,572 deaths in chickens from 11 provinces in mainland China [16]. H7N9 AIVs caused 33 human infections from the end of 2016 to 24 June 2019 [17]. Therefore, the H7N9 AIV is a zoonotic agent that poses a great threat to both public health and the poultry industry.

To better understand the differences of the pathogenicity and transmissibility between the H7N9 LPAIVs and HPAIVs, we purified and propagated two viruses, A/chicken/Guangdong/A46/2013(H7N9) (A46), which is representative of the H7N9 LPAIVs from 2013, and A/chicken/Guangdong/Q29/2017(H7N9) (Q29), which is representative of the H7N9 HPAIVs from 2017, to perform the infection experiments.

## 2. Materials and Methods

### 2.1. Viruses

The two H7N9 AIVs, A/chicken/Guangdong/A46/2013(H7N9) (A46) and A/chicken/Guangdong/Q29/2017(H7N9) (Q29), were isolated from swabs of chickens in live-bird markets in Guangdong during 2013 and 2017, respectively. These viruses were propagated using 9 to 10-day-old embryonated specific-pathogen-free (SPF) hen eggs through two-to-three rounds of the limiting dilution method [18,19]. We then collected the viruses from the allantoic fluids of multiple eggs and stored them at −80 °C. All viral experiments were performed in Biosafety Level 3 (BSL-3) facilities.

### 2.2. Animal Experiment Design

To investigate the pathogenicity and transmission of the H7N9 AIVs, A46 and Q29, in chickens, we designed our animal experiment according to previous studies [20,21]. We randomly divided 32 3- to 4-week-old SPF chickens into two groups, the A46 group and the Q29 group. The two groups were housed separately in two isolators. Eleven chickens in the A46 group were directly inoculated with a single dose of 6 log_10_EID_50_ of the A46 virus in a volume of 100 μL via the ocular-nasal route, and this was the inoculated A46 group. Three chickens, including dead and euthanized ones, in the inoculated A46 group were dissected to collect the tissues at 1 and 3 days post-infection (dpi), respectively. The remaining five chickens were observed daily for clinical symptoms and mortality until 14 dpi or until all of them died of viral infection. Five chickens in the A46 group were inoculated with 100 μL phosphate buffered saline (PBS) via the ocular-nasal route and co-housed with the inoculated A46 group to assess the transmission efficiency between the inoculated chickens and contact chickens, and this was the contact A46 group. Sixteen chickens from the Q29 group were treated and grouped in the same way as above. In addition, we raised 11 3- to 4-week-old SPF chickens alone as the control group. All control chickens were inoculated with 100 μL PBS via the ocular–nasal route and were observed for clinical symptoms and mortality for 14 days.

### 2.3. Collection of Tissues and Swab Specimens from Chickens

The chickens’ tissues including brain, kidneys, livers, lungs, spleen, trachea, ileum, bursa of Fabricius, and pancreas were collected from three chickens that were dissected randomly from the dead and euthanized chickens in the inoculated A46 and Q29 group at 1 and 3 dpi to assess the virus replication, respectively. Oropharyngeal and cloacal swabs were collected from all the chickens every other day to detect viral shedding until 13 days or until all the chickens were dead. All samples were homogenized in 1 mL of cold PBS and titrated for virus infectivity in 9- to 10-day-old SPF embryonated hen eggs. Virus titers were calculated using the Reed and Muench method [22].

### 2.4. Ethics Statements

All experiments were carried out in ABSL-3 facilities in compliance with approved protocols (SCAUABSL2017-006) by the biosafety committee of South China Agriculture University. All animals were handled in accordance with the approved guidelines of the Experimental Animal Administration and Ethics Committee of South China Agriculture University (SCAUABSL2017-006; 16 May, 2017).

## 3. Results

### 3.1. Pathogenicity of H7N9 AIVs in Chickens

**Figure 1:**
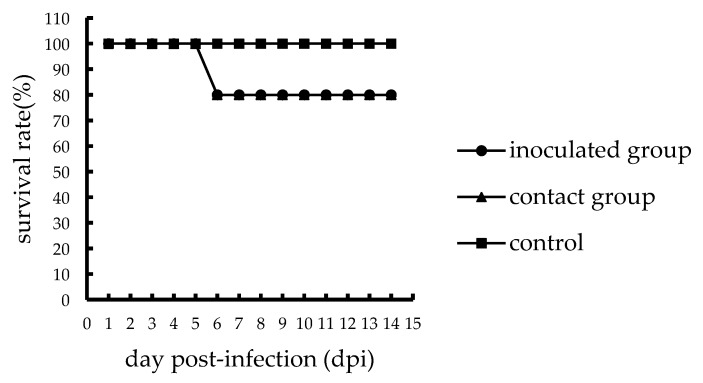
Survival of specific-pathogen-free (SPF) chickens infected with the A46 virus.

**Figure 2:**
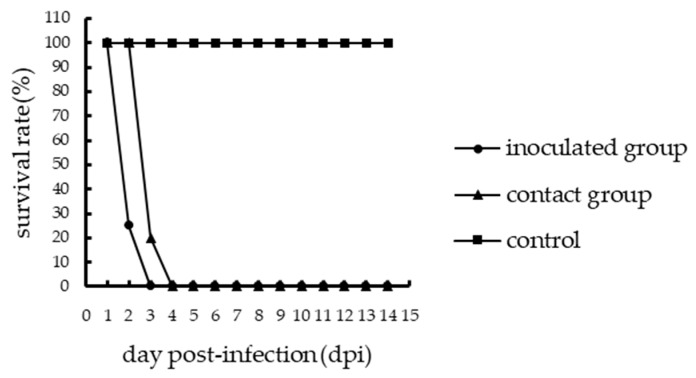
Survival of SPF chickens infected with the Q29 virus.

One inoculated chicken in the A46 group showed mild clinical symptoms such as depression and ruffled feathers, and one died of viral infection at 6 dpi; thus, the mortality of the inoculated A46 chickens was 1/5 (Figure 1). However, the inoculated chickens of the Q29 group showed severe clinical symptoms such as lethargy, mental sluggishness, and dyspnea, and all the five chickens died of viral infection within 3 days. Thus, the mortality of the inoculated Q29 chickens was 5/5 (Figure 2). Therefore, the Q29 virus caused a higher mortality in inoculated chickens than the A46 virus.

To assess the replication of H7N9 AIVs in the inoculated chickens, the brain, kidneys, livers, lungs, spleen, trachea, ileum, bursa of Fabricius, and pancreas were collected at 1 dpi and 3 dpi, respectively. In the inoculated A46 group, virus was detected in the kidneys, livers, lungs, trachea, and pancreas, with mean virus titers of 1.58–3.17 log_10_EID_50_ at 1 dpi. The virus was detected in all collected tissues, and the mean virus titers were 1.75–4.92 log_10_EID_50_ at 3 dpi (Table 1). In the inoculated Q29 group, virus was detected in all collected tissues except the spleen and pancreas, and the mean virus titers were 4.25–6.58 log_10_EID_50_ at 1 dpi. The virus was detected in all collected tissues, and the mean virus titers were 3.75–8.25 log_10_EID_50_ at 3 dpi. Our results showed that Q29 viral titers in most tissues were obviously higher than those of the A46 virus (*p* < 0.05 or *p* < 0.01). Consequently, Q29 virus replication was more efficient than that of the A46 virus in inoculated chickens.

### 3.2. Viruses Shedding from the Inoculated Chickens

Oropharyngeal and cloacal swabs were collected from the inoculated chickens to detect viral shedding every other day until 13 dpi or until all the chickens died. Virus can be tested from the A46 inoculated chicken swabs at 1, 3, and 5 dpi, although four chickens survived to the end of the experiment. The virus titers in the oropharyngeal swabs were 2.22, 2.06, and 2.33 log_10_EID_50_ at 1 dpi, 3 dpi, and 5 dpi, respectively. Virus titers in the cloacal swabs were 2.38, 3.75, and 2.13 log_10_EID_50_ at 1 dpi, 3 dpi, and 5 dpi, respectively. However, Q29 inoculated chickens’ swabs were collected at 1 and 3 dpi because all the chickens died within 3 days. The virus titers in the oropharyngeal swabs were 2.38 and 2.88 log_10_EID_50_ at 1 and 3 dpi, respectively. In addition, the virus titers in the cloacal swabs were 2.21 log_10_EID_50_ at 1 dpi and 3.13 log_10_EID_50_ at 3 dpi (Table 2). In conclusion, the A46 virus and Q29 virus can shed through both respiratory and digestive routes in inoculated chickens.

### 3.3. Transmission of H7N9 AIVs in Chickens

To evaluate the transmission of the two viruses in chickens, we counted the number of chickens that died in the contact groups and collected oropharyngeal and cloacal swabs from them. In the contact A46 group, only one chicken showed mild clinical symptoms and died of viral infection at 6 dpi, while others survived until the end of the experiment. Thus, the mortality of the contact A46 chickens was 1/5 (Figure 1). In the contact Q29 group, all chickens died of viral infection within 4 days, of which four died at 3 dpi and one died at 4 dpi. Therefore, the mortality of the contact Q29 chickens was 5/5 (Figure 2). Consequently, the Q29 virus showed a higher pathogenicity than the A46 virus in contact chickens.

Throughout the observation period, the virus could be detected from the A46 contact chickens’ swabs at 3, 5, and 7 dpi and in the Q29 contact chickens’ swabs at 3 dpi. The virus titers in the A46 contact chickens’ oropharyngeal swabs and cloacal swabs were both 1.75 log_10_EID_50_ at 3 dpi, 2 and 3.5 log_10_EID_50_ at 5 dpi, and both 2.5 log_10_EID_50_ at 7 dpi, respectively. The virus titers in the Q29 contact chickens’ oropharyngeal swabs and cloacal swabs were 2.56 log_10_EID_50_ and 5.38 log_10_EID_50_ at 3 dpi, respectively (Table 2). These results indicated that both the A46 virus and the Q29 virus can be transmitted between chickens by naïve contact, but the Q29 virus had a higher pathogenicity in contact chickens compared to the A46 virus.

## 4. Discussion

In 2013, the first case of H7N9 low pathogenic influenza virus infection in a human was reported in East China. By June 13, 2016, 781 human infection cases had been diagnosed, with a mortality rate of 40.1% [23]. The H7N9 low pathogenic influenza virus was also detected in poultry and it spread across the live poultry markets, but infected poultry did not show clinical signs and did not die. In 2016, the virus had mutated and some highly pathogenic strains that were associated with a high mortality rate had emerged. Since then, the H7N9 low pathogenic influenza virus has caused over 30 human infections in China. In addition, the emerging H7N9 highly pathogenic influenza virus had caused 15 outbreaks in chickens in 11 provinces in China. Therefore, it is necessary to analyze the differences of pathogenicity and transmissibility between H7N9 low pathogenic influenza viruses and highly pathogenic influenza viruses.

Previous studies showed that the results of the pathogenicity in chickens varied because there were many factors that changed. Ku et al. demonstrated that all 4-week-old chickens that were intranasally and intratracheally infected with 9.6 log_10_EID_50_/0.1 mL of a human-origin H7N9 low pathogenic influenza virus A/Anhui/1/2013 survived, but the H7N9 virus could be detected in the lungs, tracheal, and cloacal swabs [24]. Pantin-Jackwood et al. found that none of the 9-week-old chickens that were intranasally infected with 6.0 log_10_EID_50_/0.1 mL of A/Anhui/1/2013 showed signs of disease or died and no titers could be detected in the lungs and kidneys. However, the intestine and spleen showed low virus titers, and high virus titers were detected in oropharyngeal swabs [25]. Thus, infection with the human-origin H7N9 low pathogenic influenza virus could occur through virus shedding from the upper respiratory tract and cloacae without causing death in the chickens (Tables S8–S10).

Chowdhury et al. demonstrated that all the 4-week-old chickens that were intranasally inoculated with the H7N9 LPAIV (A/chicken/Tennessee/17-007431-3/2017) were healthy without exhibiting any clinical signs and the virus was only detected in the trachea. Shedding of the virus was only detected in oral swab samples at 3 dpi, indicating that viral replication was limited to the upper respiratory tract [26]. Zhang et al. showed that no clinical symptoms were seen in the 6-week-old chickens that were intravenously inoculated or the 3-week-old chickens that were intranasally inoculated with a H7N9 LPAIV (A/chicken/Shanghai/S1053/2013). But the virus could be detected in tracheal and cloacal swabs of the chickens inoculated intranasally with 6 log_10_EID_50_ of the virus [12]. Jacob et al. found that viral RNA could be detected by PCR in the lungs and brain from chickens that were inoculated with avian-origin LPAIV H5N2, H7N1, H7N7, or H9N2 at 2 or 4 dpi [27]. Jiao et al. demonstrated that chickens that were inoculated with 8 log_10_EID_50_ of two H7N9 LPAIVs in a 0.2 mL volume did not show any signs of disease, but the viruses could be detected in most organs including the brains of the inoculated chickens at 3 and 5 dpi [28]. Here, we found that one of five A46-inoculated chickens died of viral infection and the H7N9 LPAIV could be detected in the brain, kidneys, livers, lungs, spleen, trachea, ileum, bursa of Fabricius, pancreas, oropharynx, and cloaca. The possible reasons why the A46 inoculated chicken died and why the virus could be detected in most of its organs are as follows: (1) the 3- to 4-week-old chickens we selected were younger than the 6-week-old chickens selected in some previous studies [12,28], (2) the inoculation dose was high, and (3) the differences in amino acids between A46 and other H7N9 LPAIVs (Tables S1–S7). These results indicate that some avian-origin H7N9 LPAIVs could replicate in infected chickens’ lungs and brain when the chickens were inoculated with the higher dose, suggesting that these viruses can pass through the blood–brain barrier under certain experimental conditions (Tables S8–S10).

Shi et al. found that all 6-week-old chickens intranasally inoculated with 6 log_10_EID_50_/0.1 mL of A/chicken/Guangdong/SD008/2017 (H7N9 HPAIV) died within 4 days. The virus replicated systemically in chickens and was detected in both pharyngeal and cloacal swabs [29]. Wang et al. demonstrated that A/chicken/Guangdong/Q1/2016 (Q1), A/chicken/Guangdong/Q26/2017 (Q26) and A/chicken/Guangdong/Q39/2017 (Q39) could be replicated systemically in chickens that were inoculated with 6 log_10_EID_50_/0.1 mL of these H7N9 HPAIVs, causing all the infected chickens to die [15]. Here, we found that all chickens that were inoculated with the HPAIV Q29 via the ocular-nasal route were dead within 3 days and the virus could be detected in all collected tissues and swabs. These results indicate that the H7N9 HPAIV of avian origin could cause rapid death in chickens, replicate in several tissues, and shed from the oropharynx and cloaca. Generally, the pathogenicity, replication, and shedding of H7N9 HPAIVs in chickens are more effective than those of H7N9 LPAIVs (Tables S8–S10).

Numerous studies have shown that different H7N9 viruses exhibit different transmissibility. When 8-week-old chickens were co-housed with the chickens that were oculonasally inoculated with 10^6^ TCID_50_ (50% tissue culture infective doses) of the A/Anhui/1/2013 (H7N9), the contact chickens could shed the virus through the respiratory route at 2 to 8 days postexposure, indicating successful transmission of the virus [30]. However, when 5 log_10_EID_50_ of the A/Anhui/1/2013 (H7N9) was used to intranasally inoculate chickens, Vidaña et al. found that no viral shedding was tested in the oropharyngeal and cloacal swabs of the contact chickens [31]. The above results indicate that a higher infected dose of human-origin H7N9 influenza virus is helpful for viral shedding and transmission in chickens [32]. Jiao et al. placed 6-week-old chickens in contact with the groups that were inoculated intranasally with 8 log_10_EID_50_ of the LPAIVs A/chicken/Guangdong/110/2013 and A/chicken/Guangdong/134/2013. They observed that no contact chickens showed obvious symptoms during the observation period, but both viruses were present in the oropharyngeal and cloacal swabs from the contact chickens [28]. In this study, we found that the A46 virus could be transmitted between chickens by naïve contact and cause death in the contact chickens. These studies confirmed that H7N9 LPAIV of avian origin could be transmitted in chickens through direct contact. Wang et al. also demonstrated that the H7N9 HPAIVs, Q1, Q26, and Q39 could cause the contact chickens to die within 7, 4, and 5 days, respectively, and the virus titers in lung samples for each contact group were 7.5, 8.0, and 8.1 log_10_EID_50_/g/0.1 mL, respectively [15]. Our results showed that the Q29 virus could be transmitted among chickens, cause all the contact chickens to die within 4 days, and be shed from the contact chickens’ oropharynx and cloacae. Thus, chickens that are in contact with H7N9 HPAIV-infected chickens could become sick and die, and H7N9 HPAIV has higher pathogenicity in contact chickens compared to H7N9 LPAIV (Tables S8 and S11).

In summary, both the early and emerging H7N9 AIVs could be detected in infected chickens, and the infected chickens initiated viral shedding to naïve contact chickens through respiratory and digestive routes. However, compared with the early H7N9 LPAIVs, the pathogenicity and transmissibility of the emerging H7N9 HPAIVs are stronger in chickens, suggesting that the H7N9 influenza virus may continue to threaten human and poultry health.

## Figures and Tables

**Table 1:** Characteristics of the novel avian influenza (H7N9) viruses isolated from chickens ^a,b^.

Strains	Titer (log_10_EID_50_)	Time ^d^	Virus Replication (log_10_EID_50_/0.1mL) ^c^ in								
			Brain	Kidneys	Livers	Lungs	Spleen	Trachea	Ileum	Bursa of Fabricius	Pancreas
A46	8	1 dpi	<1.5	2.08 ± 0.29	1.58 ± 0.14	3.17 ± 1.38	<1.5	3 ± 1.3	<1.5	<1.5	1.67 ± 0.14
		3 dpi	2 ± 0.43	3.5 ± 1.73	1.83 ± 0.38	4.92 ± 1.61	1.75 ± 0.43	4.08 ± 0.72	2.83 ± 2.31	2.5 ± 1	2.08 ± 1.01
Q29	8.32	1 dpi	5.67 ± 0.72 ^e^	6.58 ± 1.04 ^e^	4.25 ± 0.5 ^e^	6.42 ± 0.76 ^f^	<1.5	5.25 ± 1	5.08 ± 0.76 ^e^	4.33 ± 0.52 ^e^	<1.5
		3 dpi	7.5 ± 0.75 ^e^	8.25 ± 1.56 ^f^	7.25 ± 0.5 ^e^	8.17 ± 1.15 ^f^	4.75 ± 0.66 ^e^	6.67 ± 1.01 ^f^	3.75 ± 1.56	6.83 ± 0.58 ^e^	5.58 ± 0.88 ^f^

^a^ 11 3- to 4-week-old SPF chickens were inoculated intranasally (i.n.) with 6 log_10_EID_50_ of the A46 virus in a volume of 100 μL PBS and 5 chickens with nothing as the contact group; three inoculated chickens were dissected randomly from euthanized chickens in the inoculated A46 group and their tissues were collected on 1dpi) and 3 dpi to detect replication of viruses in tissues. ^b^ 11 3- to 4-week-old SPF chickens were inoculated intranasally (i.n.) with 6 log_10_EID_50_ of the Q29 virus in a volume of 100 μL PBS and 5 chickens with nothing as the contact group; three inoculated chickens were dissected randomly from the dead and euthanized chickens in the inoculated Q29 group and their tissues were collected on 1 dpi and 3 dpi to detect replication of viruses in tissues. ^c^ For statistical analysis, a value of 1.5 was assigned if the virus was not detected from the undiluted sample in three embryonated hen eggs. Virus titers are expressed as means standard deviation in log_10_EID_50_/0.1 mL of tissue. ^d^ day post-infection (dpi) ^e^ *p* value was 0.05 compared with the titers in the corresponding organs of the A46 inoculated chickens. ^f^ *p* value was 0.01 compared with the titers in the corresponding organs of the A46 inoculated chickens.

**Table 2:** Virus titers in cloacal and oropharyngeal swabs from SPF chicken.

Days Post-Inoculation (log_10_EID_50_/0.1mL) ± SD ^a^																
Host	Strains		11 dpi ^f^		3 dpi		5 dpi		7 dpi		9 dpi		11 dpi		13 dpi	
			Oro ^e^	Clo ^e^	Oro.	Clo.	Oro.	Clo.	Oro.	Clo.	Oro.	Clo.	Oro.	Clo.	Oro.	Clo.
CK ^d^	A46	Ino ^b^	2.22 ± 0.57	2.38 ± 0.18	2.06 ± 0.38	3.75	2.33 ± 0.14	2.13 ± 0.53	0/4	0/4	0/4	0/4	0/4	0/4	0/4	0/4
			8/11	2/11	4/8	1/8	3/5	2/5								
		Con ^c^	0/5	0/5	1.75	1.75	2.0 ± 0.35	3.5	2.5	2.5	0/4	0/4	0/4	0/4	0/4	0/4
					1/5	1/5	2/5	1/5	1/4	1/4						
	Q29	Ino.	2.38 ± 0.18	2.21 ± 0.25	2.88 ± 0.53	3.13 ± 0.53	-	-	-	-	-	-	-	-	-	-
			2/11	6/11	2/2	2/2										
		Con.	0/5	0/5	2.56 ± 0.24	5.38 ± 0.18	-	-	-	-	-	-	-	-	-	-
					4/5	2/5										

^a^ For statistical purposes, a value of 1.5 was assigned if the virus was not detected from the undiluted sample in three embryonated hen’s eggs. ^b^ Chickens inoculated with virus. ^c^ Chickens not treated. ^d^ Host was chicken (CK). ^e^ Oropharyngeal swabs (Oro.) and cloacal swabs (Clo.) ^f^ Days post-infection (dpi).

## Supplementary Materials

The following are available online at https://www.mdpi.com/1999-4915/11/11/1047/s1, Table S1: Different amino acids in the HA gene of the chicken LPAIV H7N9 (A46), the human LPAIV H7N9 and Tennessee/H7N9; Table S2: Different amino acids in the NA gene of the chicken LPAIV H7N9 (A46), the human LPAIV H7N9 and Tennessee/H7N9; Table S3: Different amino acids in the PB1 genes of the chicken LPAIV H7N9 (A46), the human LPAIV H7N9 and Tennessee/H7N9; Table S4: Different amino acids in the PB2 genes of the chicken LPAIV H7N9 (A46), the human LPAIV H7N9 and Tennessee/H7N9; Table S5: Different amino acids in the NP genes of the chicken LPAIV H7N9 (A46), the human LPAIV H7N9 and Tennessee/H7N9; Table S6: Different amino acids in the NS1 genes of the chicken LPAIV H7N9 (A46), the human LPAIV H7N9 and Tennessee/H7N9; Table S7: Different amino acids in the M genes of the chicken LPAIV H7N9 (A46), the human LPAIV H7N9 and Tennessee/H7N9; Table S8: Our study on the pathogenicity and transmissibility of AIVs; Table S9: Studies on the pathogenicity of H7N9 influenza viruses; Table S10: Studies on the pathogenicity of LPAIVs in brain; Table S11: Studies on the transmissibility of H7N9 influenza viruses.
