# Supplementary material for: Comparative Pathogenicity and Transmissibility of the H7N9 Highly Pathogenic Avian Influenza Virus and the H7N9 Low Pathogenic Avian Influenza Virus in Chickens
Source: Viruses. 2019 Nov 10;11(11):1047. doi: 10.3390/v11111047 (PMC6893717; doi:10.3390/v11111047)
Supplement: Supplementary file 1 [file viruses-11-01047-s001.pdf]

Supplementary Materials for

Comparative Pathogenicity and Transmissibility of the H7N9 Highly Pathogenic Avian Influenza Virus and the H7N9 Low Pathogenic Avian Influenza Virus in Chickens

Hao Yu[1, †], Kunpeng Zhang[1, †], Xumeng Ye[1, †], Wenqing Wang[1], Wenbo Wu[1], Xia Wang[1], Yun Guan[1], Zhuoliang He[1], Yong Wang [2,*] and Peirong Jiao [1,*]

[1] College of Veterinary Medicine, South China Agricultural University, 483 Wushan Road, Tianhe District, Guangzhou 510642, China; yh_yuhao@yeah.net (H.Y.); kunpzhang@sohu.com (K.Z.); yexumeng@yeah.net (X.Y.); wwq201909@sina.com (W.Wang); wenbo0816@foxmail.com (W.Wu); wangxia1021@outlook.com (X.W); gguany@sina.com (Y.G.); hezhuoliang@foxmail.com (Z.H.)

[2] Department of Epidemiology, Public Health College, Harbin Medical University, Harbin 150081, China; nmndwy@163.com (Y.W.)

* Correspondence: nmndwy@163.com (Y.W.); prjiao@scau.edu.cn (P.J.); Tel.: +86-020-85283309 (P.J.)

† These authors contributed equally to this article.

This PDF file includes:

Tables S1 to S11

Table S1. Different amino acids in the HA gene of the chicken LPAIV H7N9 (A46), the human LPAIV H7N9 and chicken Tennessee/H7N9.

Virus	Amino acid sequence															
	**7-13**	**15-18**	**29**	**42**	**53-54**	**57-58**	**61-62**	**64-67**	**78-79**	**91**	**102**	**111**	**115**	**122**	**130**	**150-151**
A/chicken/Guangdong/A46/2013	VFALIAI	PTNA	S	V	RT	PR	SK	KRTV	IT	S	S	V	A	E	A	SG
A/Anhui/1/2013	VFALIAI	PTNA	S	V	RT	PR	SK	KRTV	IT	S	S	V	A	E	A	SG
A/chicken/Tennessee/17-007431-3/2017	ALIACML	GAKG	A	I	TA	KK	TQ	RKPT	LI	D	T	T	S	G	S	LD

Virus	Amino acid sequence															
	**165-166**	**178**	**180**	**182-183**	**187**	**191**	**195**	**197-198**	**211**	**217**	**223**	**235**	**245**	**247**	**255**	**272**
A/chicken/Guangdong/A46/2013	TD	K	T	KS	V	I	V	TA	V	N	V	L	M	N	S	K
A/Anhui/1/2013	TD	K	T	KS	V	I	V	TA	V	N	V	L	M	N	S	K
A/chicken/Tennessee/17-007431-3/2017	SN	R	P	SK	I	V	G	AD	I	K	T	Q	L	D	T	E

Virus	Amino acid sequence															
	**274**	**276**	**279**	**281-282**	**284-285**	**287**	**291**	**293**	**298**	**300**	**307-308**	**310**	**321**	**330**	**335**	**338**
A/chicken/Guangdong/A46/2013	M	I	G	QV	AN	E	Y	S	I	N	DS	S	R	K	I	G
A/Anhui/1/2013	M	I	G	QV	AN	E	Y	S	I	N	DS	A	R	K	I	G
A/chicken/Tennessee/17-007431-3/2017	L	V	D	PL	SS	K	F	N	V	S	NP	T	T	R	N	T

Virus	Amino acid sequence															
	**373**	**394**	**396**	**410**	**412**	**414**	**426-427**	**447**	**450**	**454-455**	**462-463**	**486**	**489**	**499-500**	**503**	**505-506**
A/chicken/Guangdong/A46/2013	N	V	E	N	V	K	FI	N	G	KD	KR	D	A	SK	E	AM
A/Anhui/1/2013	T	L	E	N	V	K	SI	I	A	MD	KR	D	A	SK	E	AM
A/chicken/Tennessee/17-007431-3/2017	T	L	D	S	I	Q	SM	I	A	MN	RK	Q	E	TQ	A	SL

Virus	Amino acid sequence				
	**517**	**524**	**536**	**541**	**549**
A/chicken/Guangdong/A46/2013	I	V	I	V	V
A/Anhui/1/2013	L	V	I	V	V
A/chicken/Tennessee/17-007431-3/2017	L	I	L	A	I

Table S2. Different amino acids in the NA gene of the chicken LPAIV H7N9 (A46), the human LPAIV H7N9 and chicken Tennessee/H7N9.

Virus	Amino acid sequence															
	**16**	**19**	**40**	**45**	**58**	**51**	**53**	**69-73**	**80-82**	**84**	**112**	**175**	**183**	**189-190**	**207**	**215**
A/chicken/Guangdong/A46/2013	I	A	G	H	P	T	T	XXXXX	RTS	N	S	V	T	KS	V	A
A/Anhui/1/2013	I	A	G	H	P	T	T	XXXXX	RTS	N	S	V	T	KS	V	A
A/chicken/Tennessee/17-007431-3/2017	V	T	N	N	S	S	A	QISNT	KAN	E	N	I	A	RA	I	T

Virus	Amino acid sequence															
	**222**	**238**	**247**	**253**	**256**	**269**	**285-287**	**334-335**	**343**	**354**	**359**	**361**	**371**	**373**	**375**	**392**
A/chicken/Guangdong/A46/2013	Q	V	P	D	I	S	ERT	NI	S	L	G	N	L	F	F	L
A/Anhui/1/2013	R	V	S	D	I	S	ERT	NI	P	S	A	T	S	S	Y	I
A/chicken/Tennessee/17-007431-3/2017	R	I	S	E	V	P	VQA	AV	P	S	G	T	S	S	Y	T

Virus	Amino acid sequence											
	**401**	**406**	**409**	**413**	**415**	**418**	**422**	**428**	**446-447**	**450**	**459**	**463**
A/chicken/Guangdong/A46/2013	G	F	F	F	G	E	G	V	GL	F	K	W
A/Anhui/1/2013	A	Y	S	Y	A	D	A	L	VS	S	N	G
A/chicken/Tennessee/17-007431-3/2017	T	Y	S	Y	A	E	A	L	VS	S	N	G

Table S3. Different amino acids in the PB1 genes of the chicken LPAIV H7N9 (A46), the human LPAIV H7N9 and chicken Tennessee/H7N9.

Virus	Amino acid sequence															
	**13-14**	**48**	**59**	**113**	**172**	**257**	**368**	**375**	**383**	**387**	**394**	**430**	**525**	**573**	**628**	**638**
A/chicken/Guangdong/A46/2013	PV	K	T	I	D	A	V	N	K	E	P	K	V	A	M	D
A/Anhui/1/2013	PV	K	T	I	D	A	V	N	K	E	P	K	V	A	M	D
A/chicken/Tennessee/17-007431-3/2017	KA	Q	S	V	E	T	I	S	E	K	H	R	I	S	L	E

Table S4. Different amino acids in the PB2 genes of the chicken LPAIV H7N9 (A46), the human LPAIV H7N9 and chicken Tennessee/H7N9.

Virus	Amino acid sequence													
	8	191	292	389	392	473	477	508	559	566	598	627	648	676
A/chicken/Guangdong/A46/2013	R	K	V	K	Q	V	V	R	N	Q	V	E	V	M
A/Anhui/1/2013	R	K	V	K	Q	M	V	R	N	Q	V	K	V	M
A/chicken/Tennessee/17-007431-3/2017	K	E	I	R	H	M	I	Q	T	H	T	E	L	T

Table S5. Different amino acids in the NP genes of the chicken LPAIV H7N9 (A46), the human LPAIV H7N9 and chicken Tennessee/H7N9.

Virus	Amino acid sequence													
	**16**	**34**	**52**	**77**	**105**	**136**	**186**	**352**	**373**	**377**	**406**	**450**	**478**	**482**
A/chicken/Guangdong/A46/2013	G	S	N	R	V	L	I	M	A	N	V	S	S	N
A/Anhui/1/2013	G	S	N	R	V	L	I	M	A	N	V	S	S	N
A/chicken/Tennessee/17-007431-3/2017	S	G	Y	K	M	M	V	V	T	S	I	N	F	S

Table S6. Different amino acids in the NS1 genes of the chicken LPAIV H7N9 (A46), the human LPAIV H7N9 and chicken Tennessee/H7N9.

Virus	Amino acid sequence														
	**27**	**47**	**55**	**60**	**63-64**	**77**	**80**	**87**	**103**	**106**	**111-112**	**124**	**139**	**143**	**172**	**179-180**
A/chicken/Guangdong/A46/2013	M	S	R	E	HM	F	S	p	L	I	IT	V	N	A	K	EI
A/Anhui/1/2013	M	S	R	E	HI	F	S	p	L	I	IT	V	N	A	K	EI
A/chicken/Tennessee/17-007431-3/2017	L	G	E	A	QI	L	T	S	F	M	VA	W	D	T	E	GV

Virus	Amino acid sequence															
	**185**	**193**	**207**	**212**	**215-216**	**218**	**221**	**223**	**226-228**	**231**	**236**	**241**	**244**	**246-249**	**251**	**253**
A/chicken/Guangdong/A46/2013	F	R	D	S	ST	-	E	E	VKP	R	V	T	I	NYGE	L	A
A/Anhui/1/2013	F	R	D	S	ST	-	E	E	VKP	R	V	T	I	NYGE	L	A
A/chicken/Tennessee/17-007431-3/2017	L	Q	N	P	PP	Q	K	A	IES	-	A	A	T	DHRK	F	T

Virus	Amino acid sequence	
	**256**	**275**
A/chicken/Guangdong/A46/2013	F	V
A/Anhui/1/2013	F	V
A/chicken/Tennessee/17-007431-3/2017	I	I

Table S7. Different amino acids in the M genes of the chicken LPAIV H7N9 (A46), the human LPAIV H7N9 and chicken Tennessee/H7N9.

Virus	Amino acid sequence															
	**15**	**37**	**46**	**95**	**101**	**107**	**140**	**142**	**144**	**157**	**166**	**219**	**224**	**227**	**234**	**242**
**A/chicken/Guangdong/A46/2013**	I	A	I	K	K	M	A	G	L	A	A	V	N	T	I	N
**A/Anhui/1/2013**	I	A	I	K	K	M	A	G	L	A	A	V	N	T	I	N
**A/chicken/Tennessee/17-007431-3/2017**	V	T	L	R	R	T	T	V	F	S	V	T	S	A	L	K

Virus	Amino acid sequence									
	**248**	**254**	**256-257**	**259-262**	**265**	**284**	**297**	**300**	**306**	**326**
**A/chicken/Guangdong/A46/2013**	L	A	SR	S - HY	I	F	N	V	S	E
**A/Anhui/1/2013**	L	A	SR	S - HY	I	F	N	V	S	E
**A/chicken/Tennessee/17-007431-3/2017**	M	S	RY	RKYH	L	P	S	I	P	G

Table S8. Our study on the pathogenicity and transmissibility of AIVs.

Chickens' age	Infected chickens' inoculated doses	Infected chickens' inoculation route	Infection route of uninoculated chicken	H7N9 virus	Pathogenicity				Transmissibility			
					Mortality	Virus replication			Mortality	Virus shedding		
						Specimens	Days	Titer		Routs	Days	Titer
Three to four-week-old	6 $\log_{10}EID_{50}$	Ocular-nasal route	Direct contact	A/chicken/Guangdong /A46/2013 (LPAIV)	20%	Kidneys, livers, lungs, trachea & pancreas	1dp	1.58-3.17 $\log_{10}EID_{50}$/mL	1/5	Oropharynx	3-7 dpi	1.75-2.5 $\log_{10}$ EID_{50}/mL
						Brain, kidneys, livers, lungs, spleen, trachea, ileum, bursa of fabricius & pancreas	3dpi	1.75-4.92 $\log_{10}EID_{50}$/mL				
						Oropharynx	1-5 dpi	2.06-2.33 $\log_{10}EID_{50}$/mL		Cloacae		1.75-3.5 $\log_{10}$ EID_{50}/mL
						Cloacae		2.13-3.75 $\log_{10}EID_{50}$/mL				
				A/chicken/Guangdong /Q29/2017 (HPAIV)	100%	Brain, kidneys, livers, lungs, trachea, ileum & bursa of fabricius	1dpi	1.50-6.58 $\log_{10}EID_{50}$/mL	5/5	Oropharynx	3 dpi	2.56 $\log_{10}$ EID_{50}/mL
						Brain, kidneys, livers, lungs, spleen, trachea, ileum, bursa of fabricius & pancreas	3dpi	3.75-8.25 $\log_{10}EID_{50}$/mL				
						Oropharynx	1-3 dpi	2.38-2.88 $\log_{10}EID_{50}$/mL		Cloacae		5.38 $\log_{10}$ EID_{50}/mL
						Cloacae		2.21-3.13 $\log_{10}EID_{50}$/mL				

Table S9. Studies on the pathogenicity of H7N9 influenza viruses.

Study	H7N9 virus	Doses	Inoculation route	Chickens' age	Pathogenicity			
					Mortality	Virus replication		
						Specimens	Days	Titer
Ku et al.	A/Anhui/1/2013 (LPAIV)	9.6 log_{10} EID_{50}	Intranasal and intratracheal infection	4-week-old	0	Lungs	3 & 5 dpi	4 - 5 log_{10} EID_{50}/mL
						Oropharynx	2 days p.i.	2.5 log_{10} EID_{50}/mL
						Cloacae	1 day p.i.	3.0 log_{10} EID_{50}/mL
Pantin-Jackwood et al.	A/Anhui/1/2013 (LPAIV)	6.0 log_{10} EID_{50}	Intranasal infection	9-week-old	0	Spleen & intestine	3 dpi	Low
						Kidneys & lungs	3 dpi	0
						Oropharynx	2 & 4 dpi	6.2 log_{10} EID_{50}/mL
						Cloacae	2 & 4 dpi	3 to 4 units lower than those in oropharynx
Chowdhury et al.	A/chicken/Tennessee/17-007431-3/2017 (LPAIV)	10^7 pfu/mL	Intranasal infection	4-week-old	0	Brain, lungs & spleen	3 days p.i	0
						Oropharynx	3 dpi	Low
Zhang et al.	A/chicken/Shanghai/S1053/2013 (LPAIV)	0.1 mL of a 1:10 dilution	Intravenous infection	6-week-old	0	The IVPI value is 0		
		6.0 log_{10} EID_{50}	Intranasal infection	3-week-old		Tracheal swabs	3 & 5 dpi	1.7-3.0 $log_{10}EID_{50}$
						Cloacal swabs	5, 7 & 9 dpi	1.7-4.0 $log_{10}EID_{50}$
Shi et al.	A/chicken/Guangdong/SD008/2017 (HPAIV)	6 log_{10} EID_{50}	Intranasal infection	6-week-old	100%	Tracheal and cloacal swabs, organs, brains, lungs, kidneys, spleens, pancreas, heart, liver, and cecum	3 dpi	About 5.2 to 7.3 $log_{10}EID_{50}$/g
						Oropharynx & cloacae	Not stated	About 4.2 to 5.2 $log_{10}EID_{50}$/g
Wang et al.	A/chicken/Guangdong/Q1/2016 (HPAIV)	6 log_{10} EID_{50}	Intranasal infection	6-week-old	100%	Brain, kidneys, livers, lungs, spleen & trachea	3 dpi	4.92-7.42 $log_{10}EID_{50}$/mL
	A/chicken/Guangdong/Q26/2017 (HPAIV)				100%	Brain, kidneys, livers, lungs, spleen & trachea	3 dpi	6.75-8.42 $log_{10}EID_{50}$/mL
	A/chicken/Guangdong/Q39/2017 (HPAIV)				100%	Brain, kidneys, livers, lungs, spleen & trachea	3 dpi	6.33-7.83 $log_{10}EID_{50}$/mL

Table S10. Studies on the pathogenicity of LPAIVs in brain.

Article title	Author	LPAIV	Experimental method	Results	Can it be detected in brain?
Systemic distribution of different low pathogenic avian influenza (LPAI) viruses in chicken	Jacob Post, Eveline D de Geus et al.	H5N2, H7N1, H7N7 & H9N2	1-day-old chickens were inoculated with 0.2 mL (2×10^5 EID_{50}) of the LPAIVs and their lungs, brain, ileums, blood, livers, kidneys, heart and spleen were collected for RNA extraction to test the systemic distribution.	Viral RNA was found by PCR in lungs, brain, intestine, peripheral blood mononuclear cells, livers, kidneys, heart and spleen from chickens infected with chicken isolated LPAIV H5N2, H7N1, H7N7 or H9N2 at 2 or 4 dpi. But H7N7 virus could not be isolated from brain, which was in agreement with the data from the PCR at 4 dpi.	H5N2, H7N1 & H9N2 can be detected.
H7N9 Avian Influenza Virus Is Efficiently Transmissible and Induces an Antibody Response in Chickens	Peirong Jiao, Yafen Song et al.	H7N9	6-week-old chickens were intranasally inoculated with 8 $log_{10}EID_{50}$ of A/chicken/Guangdong/110/2013 (CK110) and A/chicken/Guangdong/134/2013 (CK134) in a 0.2 mL volume, and their brain, spleen, kidneys, lungs, livers, intestine, heart, trachea, and pancreas were collected to detect the virus.	None of the chickens showed any signs of disease, and all survived until the end of the experiment. Viruses can be detected in most organs of the inoculated chickens including brains at 3 and 5 dpi. In the brain, CK110 and CK134 replicated to mean titers of 1.58–2.08 $log_{10}EID_{50}$/mL at 3 dpi and 1.83–1.92 $log_{10}EID_{50}$/mL at 5 dpi.	yes

Table S11. Studies on the transmissibility of H7N9 influenza viruses.

Study	H7N9 virus	Infected chickens' inoculated doses	Infected chickens' inoculation route	Infection route of uninoculated chicken	Chickens' age	Transmissibility		
						Mortality	Virus shedding	
							Tested routs	Can it be detected?
Kalthoff et al.	A/Anhui/1/2013 ((LPAIV))	10^6 $TCID_{50}$	Oculonasal infection	Direct contact	8-week-old	0	Respiratory route	Yes
B. Vidaña et al.	A/Anhui/1/2013 (LPAIV)	5 $log_{10}EID_{50}$	Intranasal infection	Direct contact	2-week-old	0	Oropharyngeal & cloacal swabs	No
Jiao et al.	A/chicken/Guangdong/110/2013 (LPAIV)	8 $log_{10}EID_{50}$	Intranasal infection	Direct contact	6-week-old	0	Oropharyngeal & cloacal swabs	Yes
	A/chicken/Guangdong/134/2013 (LPAIV)					0	Oropharyngeal & cloacal swabs	Yes
Wang et al.	A/chicken/Guangdong/Q1/2016 (HPAIV)	6 $log_{10}EID_{50}$	Intranasal infection	Direct contact	6-week-old	100%	Lungs	Yes
	A/chicken/Guangdong/Q26/2017 (HPAIV)					100%		Yes
	A/chicken/Guangdong/Q39/2017 (HPAIV)					100%		Yes
